# Lymphocyte Activation Gene 3 (Lag3) Contributes to α-Synucleinopathy in α-Synuclein Transgenic Mice

**DOI:** 10.3389/fncel.2021.656426

**Published:** 2021-03-10

**Authors:** Hao Gu, Xiuli Yang, Xiaobo Mao, Enquan Xu, Chen Qi, Haibo Wang, Saurav Brahmachari, Bethany York, Manjari Sriparna, Amanda Li, Michael Chang, Pavan Patel, Valina L. Dawson, Ted M. Dawson

**Affiliations:** ^1^Neuroregeneration and Stem Cell Programs, Institute for Cell Engineering, Johns Hopkins University School of Medicine, Baltimore, MD, United States; ^2^Department of Neurology, Johns Hopkins University School of Medicine, Baltimore, MD, United States; ^3^Adrienne Helis Malvin Medical Research Foundation, New Orleans, LA, United States; ^4^Department of Physiology, Johns Hopkins University School of Medicine, Baltimore, MD, United States; ^5^Solomon H. Snyder Department of Neuroscience, Johns Hopkins University School of Medicine, Baltimore, MD, United States; ^6^Department of Pharmacology and Molecular Sciences, Johns Hopkins University School of Medicine, Baltimore, MD, United States

**Keywords:** Lag3, Parkinson’ disease, α-synuclein, α-synucleinopathy, aggregation

## Abstract

Aggregation of misfolded α-synuclein (α-syn) is the major component of Lewy bodies and neurites in Parkinson’s disease (PD) and related α-synucleinopathies. Some α-syn mutations (e.g., A53T) in familial PD recapitulate the α-syn pathology in transgenic mice, which supports the importance of pathologic α-syn in driving the pathogenesis of α-synucleinopathies. Lymphocyte activation gene 3 (Lag3) is a receptor of α-syn fibrils facilitating pathologic α-syn spread; however, the role of Lag3 in mediating the pathogenesis in α-syn transgenic mice is not clear. Here, we report that depletion of Lag3 in human α-syn A53T transgenic (hA53T) mice significantly reduces the level of detergent-insoluble α-syn aggregates and phosphorylated ser129 α-syn, and inhibits activation of microglia and astrocytes. The absence of Lag3 significantly delays disease progression and reduces the behavioral deficits in hA53T transgenic mice leading to prolonged survival. Taken together, these results show that Lag3 contributes to the pathogenesis in the α-syn A53T transgenic mouse model.

## Introduction

The most common neuropathological hallmark of Parkinson’s disease (PD) and related α-synucleinopathies is the accumulation of misfolded α-synuclein (α-syn) aggregation, which is the major component of Lewy bodies and Lewy neurites (Lee and Trojanowski, 2006; Dawson et al., 2010, 2018). The discovery of α-syn mutations (A53T, A30P, and E46K) or duplication or triplication that leads to rare forms of familial PD, supports the importance of pathological α-syn in the pathogenesis of α-synucleinopathies (Polymeropoulos et al., 1997; Krüger et al., 1998; Zarranz et al., 2004; Martin et al., 2011). The α-syn transgenic mouse model overexpressing human α-syn with a familial PD-associated mutation (A53T) recapitulates α-syn pathology, which is present in α-synucleinopathies (Giasson et al., 2002; Lee et al., 2002, 2012; Lee and Trojanowski, 2006; Dawson et al., 2010; Deng and Yuan, 2014). Human α-syn A53T transgenic (hA53T) mice exhibit progressive neurodegenerative phenotypes including hyperactivity in the early stage (∼5 months), substantial α-syn pathology and glial activation at the onset of paralysis (∼9 months) followed by death (9–12 months) (Lee et al., 2002; Brahmachari et al., 2016). However, the regulator driving α-synucleinopathies is largely unexplored.

Lymphocyte-activation gene 3 (Lag3, also known as CD223) is an immune checkpoint inhibitory receptor, which is a cancer immunotherapy target (Anderson et al., 2016; Andrews et al., 2017). We have recently identified Lag3 as a receptor that mediates pathologic α-syn cell-to-cell transmission (Mao et al., 2016). Further studies of a specific single nucleotide polymorphisms (SNPs) of the Lag3 gene and the level of soluble Lag3 that are associated with PD, support Lag3 contributing to the pathogenesis of α-synucleinopathies. Since hA53T mice gradually exhibit α-syn pathology, paralysis and death due to the overexpression of the hA53T α-syn monomer, we sought to determine if Lag3 could play a role in the pathogenesis in hA53T mice. Here we report that Lag3 contributes to the pathogenesis of α-synucleinopathy in hA53T mice. In particular, depletion of Lag3 (Lag3^–/–^) markedly reduces the neurobehavioral deficits, decreases α-syn pathology, inhibits glial activation, and ultimately extends the lifespan of hA53T mice. These findings indicate that Lag3 plays an import role in the pathogenesis of α-synucleinopathy in hA53T mice.

## Results

### Depletion of Lag3 Prolongs the Survival of hA53T Mice

**FIGURE 1:**
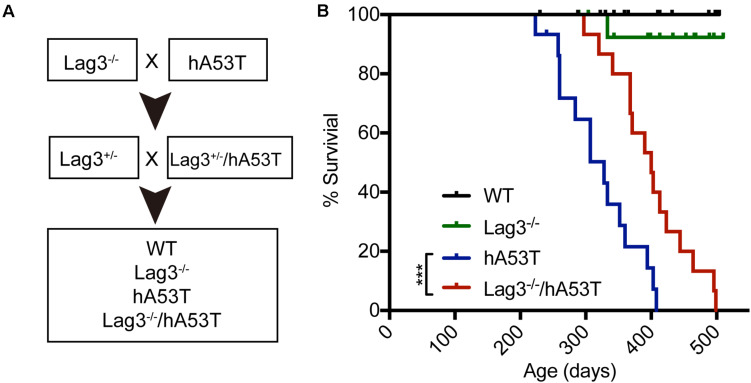
Lag3 knockout (Lag3^–/–^) prolongs survival of hA53T α-syn transgenic mice. **(A)** Breeding strategy to generate Lag3^–/–^/hA53T mice. **(B)** Kaplan-Meier survival curve analysis for WT, Lag3^–/–^, hA53T, and Lag3^–/–^/hA53T mice (*n* = 15 mice). Statistical analysis was performed with Log-rank Mantel-Cox test, ***P < 0.001. The median survival of hA53T mice is 328 days, and Lag3^–/–^/hA53T survival is prolonged to 400 days.

To test the role of Lag3 in α-syn transgenic mice, hA53T mice overexpressing hA53T α-syn under the direction of the mouse prion promoter (Lee et al., 2002) were cross-bred to the Lag3^–/–^ mice (Figure 1A). From this cross-breeding, littermates with the following genotypes were separated and aged: wildtype (WT), Lag3^–/–^, hA53T, and Lag3^–/–^/hA53T mice (Figure 1A), and survival was monitored. As previously described (Lee et al., 2002; Brahmachari et al., 2016), hA53T mice eventually developed progressive loss of the righting reflex and paralysis, and lived an average of 328 days (Figure 1B). The Lag3^–/–^/hA53T mice disease progression was delayed and they lived an average of 400 days (Figure 1B). The Kaplan-Meier survival curve significantly shows that the depletion of Lag3 prolonged the survival of hA53T mice by 72 days (Figure 1B).

### Depletion of Lag3 Alleviates Neurobehavioral Deficits

**FIGURE 2:**
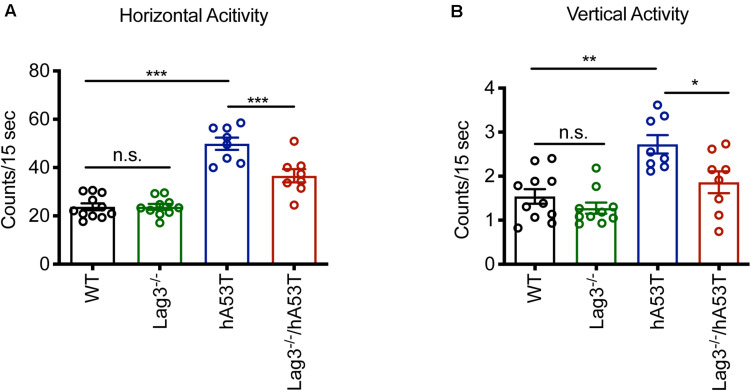
Behavioral assessment of Lag3^–/–^/hA53T mice. Novelty-induced activity was measured at 5 months of age for Lag3^–/–^/hA53T and littermate controls in an open-field test. WT (*n* = 11 mice), Lag3^–/–^ (*n* = 10 mice), hA53T (*n* = 8 mice), and Lag3^–/–^/hA53T mice (*n* = 8 mice). **(A)** Horizontal and **(B)** vertical activities were automatically recorded. Statistical significance was determined by one-way ANOVA with Turkey’s multiple comparisons test. Data are presented as the mean ± SEM. *P < 0.05, **P < 0.01, ***P < 0.001.

The hA53T mice exhibit increased hyperactivity at ∼5 months of age in the open-field test, which is thought to be the beginning of the neurodegenerative phenotype of the hA53T mice (Lee et al., 2002; Brahmachari et al., 2016). Accordingly, open-field monitoring was performed and the results show that there was no difference in the baseline behavioral test between WT and Lag3^–/–^ mice. Consistent with the prior publications (Lee et al., 2002; Brahmachari et al., 2016), the hA53T mice at ∼5 months of age exhibited increased hyperactivity (increased horizontal and vertical activities) compared to age-matched WT mice. In contrast, the depletion of Lag3 in hA53T mice reduced the hyperactivity (Figures 2A,B).

### Depletion of Lag3 Decreases α-Syn Pathology in hA53T Mice

**FIGURE 3:**
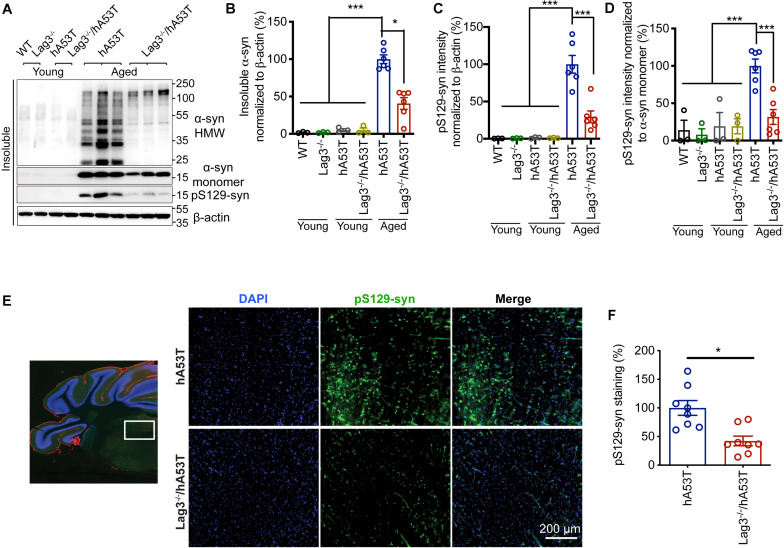
Lag3 deletion reduces α-syn pathology in hA53T mice. **(A)** Representative immunoblots of Lag3, α-syn, pS129-syn (phosphorylated ser129 α-syn), and β-actin in the detergent-insoluble fraction of the brainstem from young (2–3 months) or aged (9–12 months, symptomatic) hA53T transgenic mice and Lag3^–/–^/hA53T mice littermate controls. **(B,C)** Quantification of insoluble α-syn and pS129-syn protein levels normalized to β-actin. **(D)** Quantification of pS129-syn protein levels in a normalized to insoluble α-syn monomer. **(B–D)** Aged WT, Lag3^–/–^ mice (*n* = 3 mice per group), young hA53T and Lag3^–/–^/hA53T (*n* = 3 mice per group), aged hA53T and Lag3^–/–^/hA53T (*n* = 6 mice per group). Statistical significance was determined by one-way ANOVA with Dunnett’s multiple comparisons test. Quantified data are expressed as the mean ± SEM. *P < 0.05, ***P < 0.001. **(E)** Representative immunostaining images for pS129-syn in the brainstem highlighted in the white box of brain sections of hA53T and Lag3^–/–^/hA53T mice. Scale bar, 200 μm. **(F)** Quantification of the intensity of the pS129-syn staining (*n* = 3 mice per group). Quantified data are expressed as the mean ± SEM. Student’s *t*-test, *P < 0.05.

**FIGURE 4:**
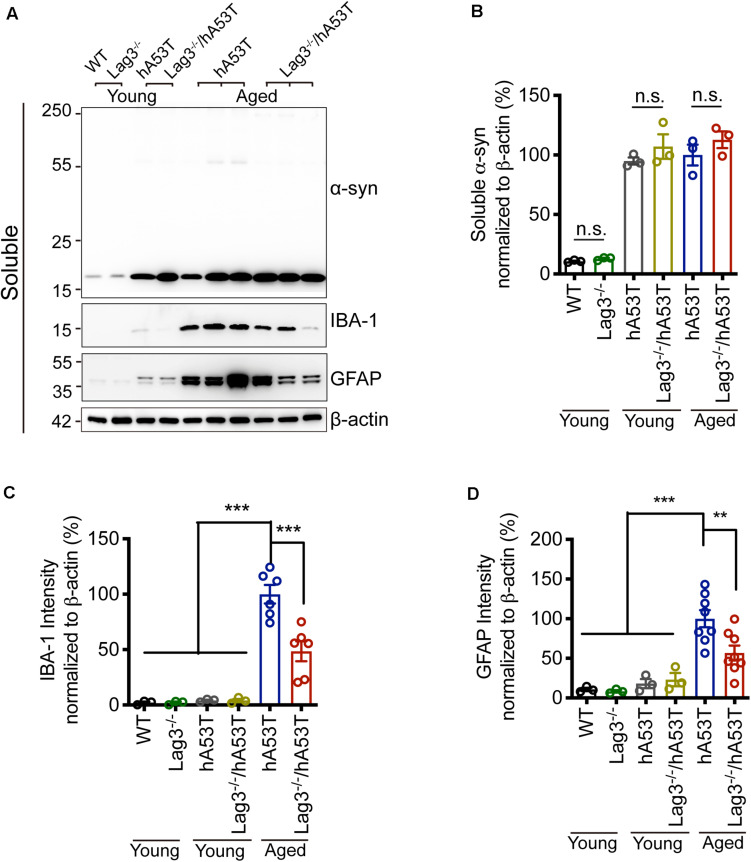
Lag3 deletion reduces glial activation in hA53T mice. **(A)** Representative immunoblots of α-syn, GFAP, IBA-1 and β-actin in the detergent-soluble fraction of the brainstem from young (2–3 months) or aged (9–12 months, symptomatic) hA53T transgenic mice and Lag3^–/–^/hA53T mice littermate controls. **(B)** Quantification of soluble α-syn normalized to β-actin. **(C,D)** Quantification of IBA-1 and GFAP normalized to β-actin. WT, Lag3^–/–^ mice, young hA53T, and young Lag3^–/–^/hA53T (*n* = 3 mice per group), aged hA53T, and aged Lag3^–/–^/hA53T (*n* = 6–8 mice per group). Statistical significance was determined by one-way ANOVA with Dunnett’s multiple comparisons test. Quantified data are expressed as the mean ± SEM. **p < 0.01, ***P < 0.001, n.s., not significance.

To determine the role of Lag3 in contributing to α-syn pathology in hA53T mice, we collected the pathologically affected brain tissue (brainstem) of aged hA53T and Lag3^–/–^/hA53T mice when they were completely paralyzed (9–12 months). We also collected the brain tissue of the age-matched WT and Lag3^–/–^ mice, and young hA53T and Lag3^–/–^/hA53T (2–3 months). We examined the levels of total α-syn and phosphorylated ser129 α-syn (pS129-syn) in the Triton X-100 insoluble fractions with immunoblots (Brahmachari et al., 2016). There was substantial detergent-insoluble α-syn aggregation and pS129-syn in aged hA53T mice (Figure 3A). In contrast, depletion of Lag3 significantly decreased the levels of detergent-insoluble α-syn aggregation and pS129-syn (Figures 3A–D). In both young hA53T and Lag3^–/–^/hA53T mice, detergent-insoluble α-syn aggregates and pS129-syn were not present (Figures 3A–D). To validate the role of Lag3 in decreasing α-syn pathology, we further assessed the immunoreactivity of pS129-syn in the pathologically affected brainstem of hA53T mice (Lee et al., 2002; Brahmachari et al., 2016). Substantial pS129-syn was observed in hA53T mice, whereas the deletion of Lag3 significantly decreased the level of pS129-syn (Figures 3E,F), which is consistent with the immunoblotting result. In the Triton X-100 soluble fraction, revealed no significant difference in the level of soluble α-syn between hA53T and Lag3^–/–^/hA53T mice (Figures 4A,B). The immunoblot results also show that depletion of Lag3 did not change the expression level of the soluble α-syn monomer.

### Depletion of Lag3 Decreases Glial Activation in hA53T Mice

Microglial activation is a reliable indicator of pathological changes in α-synucleinopathies (Bartels et al., 2020; Choi et al., 2020; Kam et al., 2020). Pathological α-syn can induce the up-regulation of ionized calcium-binding adapter molecule 1 (IBA-1) in hA53T mice, allowing discrimination between surveilling and activated microglia (Hopperton et al., 2018; Yun et al., 2018). In the brainstem of aged hA53T mice, there was a prominent increase in the expression of IBA-1 compared to young hA53T mice as evidenced by immunoblotting (Figures 4A,C). The depletion of Lag3 significantly decreased the expression of IBA-1 in hA53T mice (Figures 4A,C). We also assessed the astroglial reaction by examining the immunoreactivity of glial fibrillary acidic protein (GFAP). In the brainstem of aged hA53T, substantial GFAP immunoreactivity was observed compared to young hA53T mice (Figures 4A,D). The depletion of Lag3 significantly decreased the GFAP immunoreactivity in aged hA53T mice (Figures 4A,D).

**FIGURE 5:**
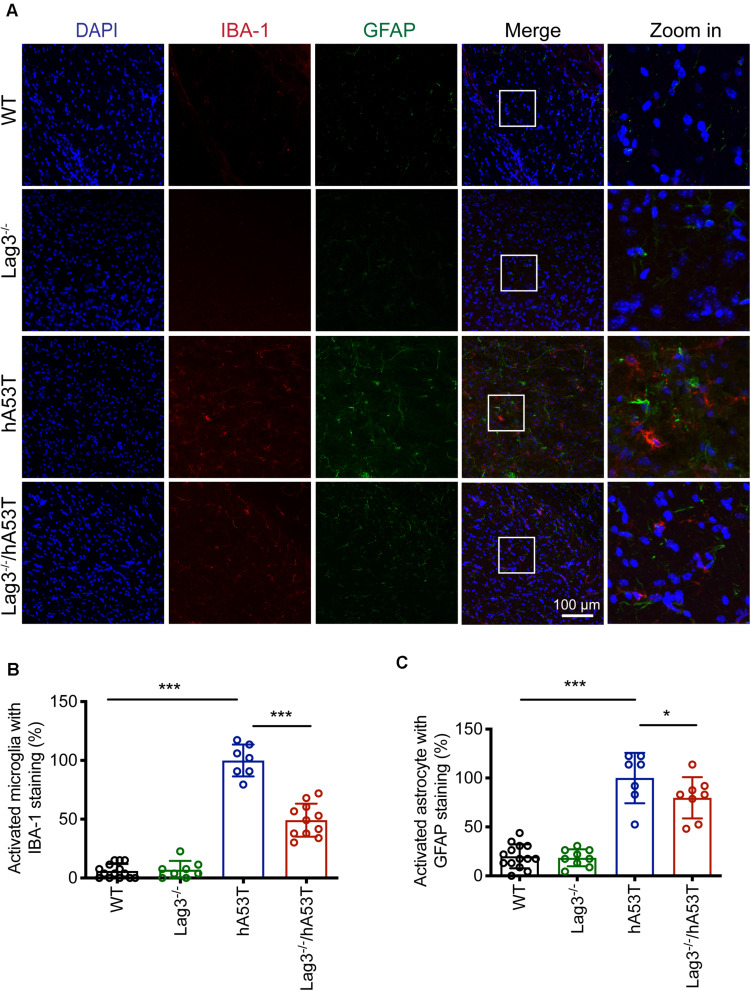
Lag3 deletion reduces glial activation in hA53T mice. **(A)** Representative immunostaining images in the brainstem for IBA-1 and GFAP in WT, Lag3^–/–^, hA53T and age matched Lag3^–/–^/hA53T aged mice. **(B,C)** Quantification of the intensity of the IBA-1 and GFAP staining in WT (4 mice), Lag3^–/–^ (5 mice), hA53T (4 mice), and Lag3^–/–^/hA53T mice (4 mice). Statistical significance was determined by one-way ANOVA with Dunnett’s multiple comparisons test. Quantified data are expressed as the mean ± SEM. *p < 0.05, ***P < 0.001, n.s., not significance. Scale bar, 100 μm.

We further examined glial activation in brain sections for IBA-1 and GFAP immunofluorescence (Figures 5A–C). The results show that aged hA53T mice exhibited substantial glial activation with up-regulated expression of IBA-1 (Figures 5A,B) and GFAP (Figures 5A,C) compared to aged WT and Lag3^–/–^ mice. The depletion of Lag3 in hA53T mice significantly inhibited the up-regulated IBA-1 and GFAP (Figures 5A–C). Taken together, these results indicate that the depletion of Lag3 decreased aggregates and pathology of insoluble α-syn, and inhibited activation of microglia and astrocytes. Ultimately, it delayed the disease progression and prolonged the survival of hA53T α-syn transgenic mice.

## Discussion

The major finding of this study is that Lag3 delays the progression of neurodegenerative disease in the hA53T α-syn transgenic mouse model. The hA53T α-syn transgenic mice overexpress hA53T α-syn protein, which leads to α-synucleinopathy throughout the central nervous system. Pathology is particularly severe in the cerebellum, brainstem and spinal cord (Lee et al., 2002). The hA53T transgenic mice exhibit severe paralysis and eventually die. Substantial α-syn pathology and significant glial activation accompany the disease progression. Before the onset of the motor impairment, hA53T α-syn transgenic mice exhibit hyperactivity on open field testing. Knockout of Lag3 significantly reduces the hyperactivity, decreases the neuropathology and glial activation of hA53T mice. This is the first report to show that deletion of Lag3 reduces neurodegeneration of α-syn transgenic mice We have identified Lag3 is an essential receptor that mediates the internalization of pathological α-syn and subsequent cell-to-cell spreading (Mao et al., 2016), the present work supports the notion that Lag3 may have other roles in contributing to neurodegeneration due to pathologic α-syn. Elucidation of the role of Lag3 in driving disease progression will require further study in the biology of Lag3 underlying the α-syn neuropathology. It will be important for understanding the pathogenesis of PD and other α-synucleinopathies.

As one of key in α-syn-related neurotoxicity, microglial activation induces reactive astrocytes by secreting inflammatory cytokines (Il-1α, TNFα, and C1q), contributing to the death of neurons in various human neurodegenerative disorders (Liddelow et al., 2017; Gelders et al., 2018; Linnerbauer et al., 2020). Microglial activation can be caused by pathological α-syn, which is an important driver in the pathogenesis of PD (Kam et al., 2018; Yun et al., 2018). MHC class II is required for α-syn-induced activation of microglia and dopaminergic neurodegeneration (Harms et al., 2013). Lag3 is a member of the immunoglobulin superfamily of receptors and is expressed in neurons, and the mRNA is found enriched in microglia (Chiu et al., 2013; Hickman et al., 2013; Zhang et al., 2014; Bennett et al., 2016). Lag3 is also a receptor for MHC II, with a binding affinity 100-fold higher than that to CD4 (Huard et al., 1995). Our biochemical and immunostaining studies suggest that the depletion of Lag3 strongly reduces microglial activation, indicating that Lag3 may can play a role in mediating neuroinflammatory response in the pathogenesis of neurodegeneration. Glia cell cross-talk is one potential pathological mechanism responsible for neuronal cell death in PD (Liddelow et al., 2017; Yun et al., 2018; Linnerbauer et al., 2020). Future studies are required to determine whether the reduction in microglial activation is due to the absence of Lag3 or a consequence of diminished neurodegeneration due to decreased cell-to-cell transmission of pathologic α-syn.

In summary, we report that Lag3 contributes to the pathogenesis of α-synucleinopathies, and depletion of Lag3 delays progression of neurodegenerative disease in hA53T α-syn transgenic mouse model. Genetic targeting of Lag3 has promise in treating α-synucleinopathies such as PD and related neurodegenerative disorders.

## Materials and Methods

### Animals

The generation of transgenic mouse overexpressing hA53T α-synuclein using the mouse prion protein (mPrP) promotor was described previously (Lee et al., 2002; Brahmachari et al., 2016). Lag3^–/–^ mice were kindly provided by Dr. Charles Drake (Johns Hopkins University), and were maintained on a C57BL6 background (Workman et al., 2004). The mice do not develop any autoimmune or inflammatory phenotype. All housing, breeding, and procedures were performed according to the NIH Guide for the Care and Use of Experimental Animals and approved by the Johns Hopkins University Animal Care and Use Committee.

### Open-Field Assay

Open-field testing was performed by placing animals into an infrared beam chamber. Activity was monitored using the Photobeam Activity System (San Diego Instruments), which provides a grid of infrared beams. The total number of beam breaks over a period of 4, 15 min intervals (total of 60 min) was recorded and analyzed.

### Tissue Lysate Preparation

After perfusion with ice-cold PBS, brains were removed and different regions including brainstem, cerebellum, spinal cord, cortex were dissected. These three brain regions were collected, mixed, and homogenized with non-ionic detergent brain lysis buffer (10 mM Tris-HCl, pH 7.4, 150 mM NaCl, 5 mM EDTA, 0.5% Nonidet P-40, Phosphatase Inhibitor Cocktail II and III (Sigma-Aldrich), and complete protease inhibitor mixture). The homogenate was centrifuged for 20 min at 4°C, 100,000*g*, and the resulting pellet (P1) and supernatant (S1) (non-ionic detergent-soluble) fractions were collected. The P1 was washed once in brain lysis buffer containing non-ionic detergent (0.5% Nonidet P-40), and then homogenized in brain lysis buffer containing 1% SDS and 0.5% sodium deoxycholate. The homogenate was centrifuged, and the resulting supernatant (S2) (non-ionic detergent-insoluble) was collected.

### Immunoblot for Mouse Brain

Protein concentrations of samples were determined using the BCA assay (Pierce, United States) and samples (10–20 μg total proteins) were loaded on SDS-polyacrylamide gels (12.5%) and transferred onto nitrocellulose membranes. Blots were blocked in 5% non-fat milk or 5% BSA in TBS-T (Tris-buffered saline, 0.1% Tween 20) and probed using various primary antibodies and related secondary antibodies, and then were detected using ECL or SuperSignal Femto substrate (Thermo Fisher Scientific, United States) and imaged by ImageQuant LAS 4000mini scanner (GE Healthcare Life Sciences, United States).

### Immunostaining for Mouse Brain

Mice were intracardially perfused with ice-cold phosphate buffered saline (PBS) and 4% paraformaldehyde (PFA)/PBS (wt/vol, pH 7.4) after anesthesia by i.p. injection of Nembutal sodium solution (50 μl of twofold dilution in PBS of pentobarbital sodium 50 mg/ml; Lundbeck). Brains were removed from mouse heads, and postfixed for 24 h in the same fixative (4% PFA). After cryoprotection in 30% sucrose/PBS (wt/vol, pH 7.4) for 2–3 days incubation, brains were cut into coronal brain sections with a microtome (with 30 μm thickness). In immunostaining procedures, brain sections were blocked with 10% donkey/goat serum (sigma/Aldrich)/PBS plus 0.3% Triton X-100, and then incubated with indicated antibodies (4° C, overnight). After three washes (10 min each) with PBS, floating brain sections were incubated with corresponding secondary antibodies conjugated with fluorescent dyes. Images were obtained using a confocal microscope (Zeiss Confocal LSM 880 with Airyscan) and digital microscope (Keyence VHX 7000, Japan).

### Quantification and Statistical Analysis

All data were analyzed using GraphPad Prism. Statistics Data are presented as the mean ± SEM with at least 3 independent experiments. Representative morphological images were obtained from at least 3 experiments with similar results. Statistical significance was assessed via a one-way ANOVA test followed by indicated *post-hoc* multiple comparison analysis. Assessments with *p* < 0.05 were considered significant.

## Data Availability Statement

The raw data supporting the conclusions of this article will be made available by the authors, without undue reservation.

## Ethics Statement

The animal study was reviewed and approved by the Johns Hopkins University Animal Care and Use Committee.

## Author Contributions

HG and XM designed the majority of the experiments, performed the experiments, analyzed the data, and wrote the manuscript. XY, EX, and HW performed the experiments and data interpretation. CQ, SB, and BY were involved in mouse husbandry. MS, AL, MC, and PP performed sample preparation and assisted with experiments. VLD, TMD, and XM supervised the project, formulated the hypothesis, designed the experiments, analyzed the data, and wrote the manuscript. All authors contributed to the article and approved the submitted version.

## Conflict of Interest

The authors declare that the research was conducted in the absence of any commercial or financial relationships that could be construed as a potential conflict of interest.
